# Supplementary material for: Hydroxychloroquine Enhances Cytotoxic Properties of Extracellular Vesicles and Extracellular Vesicle–Mimetic Nanovesicles Loaded with Chemotherapeutics
Source: Pharmaceutics. 2023 Feb 5;15(2):534. doi: 10.3390/pharmaceutics15020534 (PMC9962585; doi:10.3390/pharmaceutics15020534)
Supplement: Supplementary file 1 [file pharmaceutics-15-00534-s001.zip › pharmaceutics-2109572-supplementary.pdf]

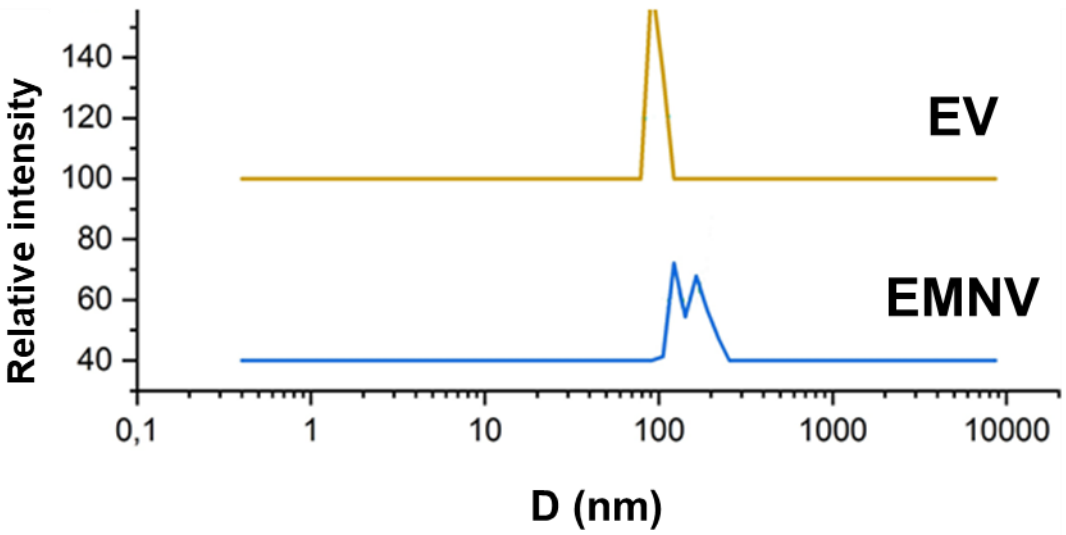

Figure S1. Analysis of nanoparticles polydispersity.

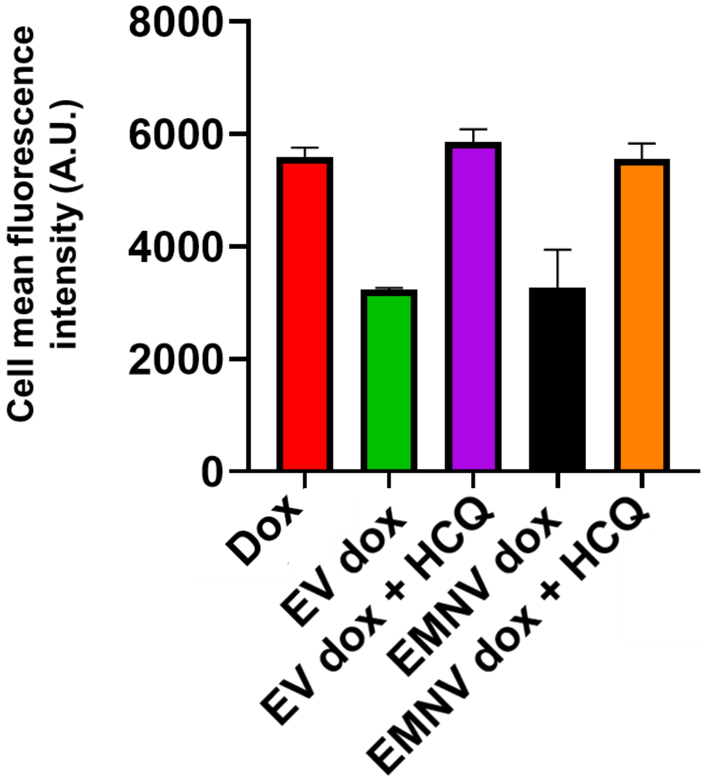

Figure S2. Flow cytometry analysis of doxorubicin mean fluorescence intensity in HEK-293T cells in different treatment groups.

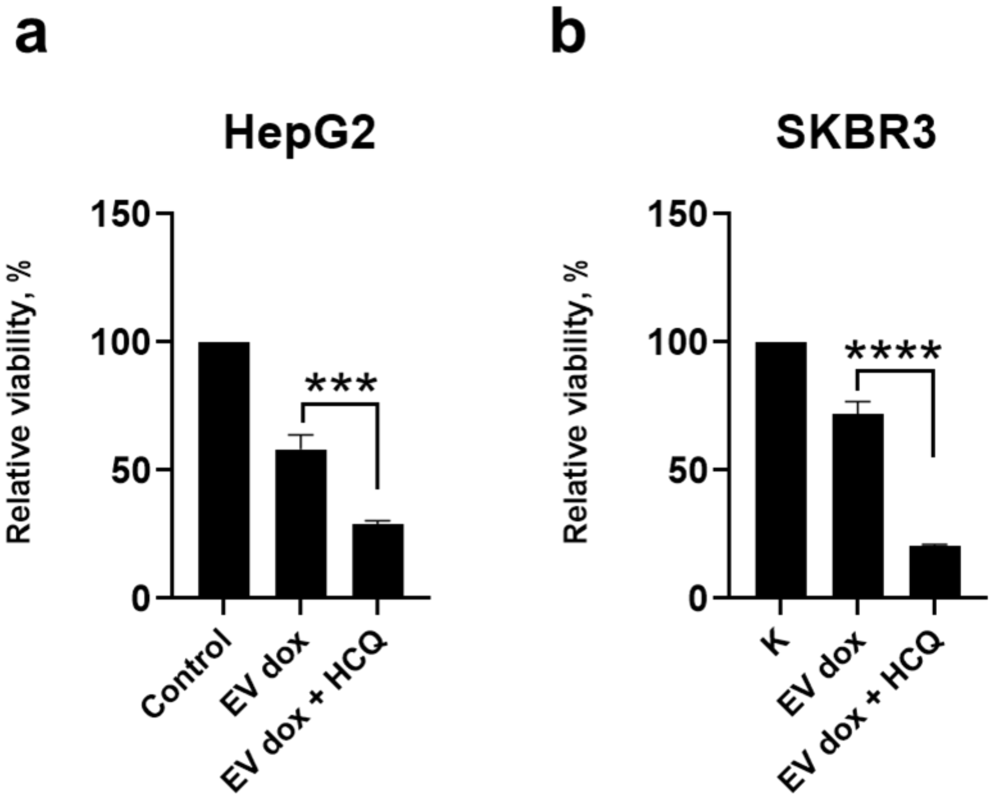

Figure S3. Viability of cancer cell lines treated with DOX-loaded EVs with and without HCQ. (a) HepG2 cells and (b) SKBR3 cells. ***p<0.001, ****p<0.0001.
